# Continuous Formulation Approaches of Amorphous Solid Dispersions: Significance of Powder Flow Properties and Feeding Performance

**DOI:** 10.3390/pharmaceutics11120654

**Published:** 2019-12-05

**Authors:** Edina Szabó, Balázs Démuth, Dorián László Galata, Panna Vass, Edit Hirsch, István Csontos, György Marosi, Zsombor K. Nagy

**Affiliations:** Department of Organic Chemistry and Technology, Budapest University of Technology and Economics (BME), Műegyetem rakpart 3, H-1111 Budapest, Hungary; szedina017@gmail.com (E.S.); demuth@oct.bme.hu (B.D.); dgalata24@gmail.com (D.L.G.); panna.vass@oct.bme.hu (P.V.); edit.hirsch@gmail.com (E.H.); icsontos@mail.bme.hu (I.C.); gmarosi@mail.bme.hu (G.M.)

**Keywords:** amorphous solid dispersions, formulation, powder characterization, feeding, continuous manufacturing

## Abstract

Preparation and formulation of amorphous solid dispersions (ASDs) are becoming more and more popular in the pharmaceutical field because the dissolution of poorly water-soluble drugs can be effectively improved this way, which can lead to increased bioavailability in many cases. During downstream processing of ASDs, technologists need to keep in mind both traditional challenges and the newest trends. In the last decade, the pharmaceutical industry began to display considerable interest in continuous processing, which can be explained with their potential advantages such as smaller footprint, easier scale-up, and more consistent product, better quality and quality assurance. Continuous downstream processing of drug-loaded ASDs opens new ways for automatic operation. Therefore, the formulation of poorly water-soluble drugs may be more effective and safe. However, developments can be challenging due to the poor flowability and feeding properties of ASDs. Consequently, this review pays special attention to these characteristics since the feeding of the components greatly influences the content uniformity in the final dosage form. The main purpose of this paper is to summarize the most important steps of the possible ASD-based continuous downstream processes in order to give a clear overview of current course lines and future perspectives.

## 1. Introduction

With the increasing number of poorly water-soluble drug candidates [1], the importance of new formulation technologies and the product development of the so-obtained materials has enhanced [2,3]. Among several strategies, applying amorphous solid dispersions (ASDs) is getting to reach more interest owing to advantageous dissolution properties of the products [4]. ASDs can significantly increase the dissolution rate and extent while in most cases the thermodynamic solubility of the active pharmaceutical ingredients (APIs) is not or just slightly changed by the matrix of the ASDs. This way the degree of supersaturation can be increased effectively, which is the main driving force of passive membrane transport [5], resulting in increased bioavailability [6,7]. However, achieving stabile forms and scaling-up of the preparation technologies may be challenging, thus these drawbacks make it more difficult to bring ASD-containing medicines to the market [8,9]. In spite of these difficulties, 14 ASD products have been approved by the Food and Drug Administration (FDA) until 2012 and further 10 ASD-loaded medicines were accepted by the FDA until 2017. Therefore, the still-growing tendency indicates a promising future of this formulation method [10]. With this in mind, the next question is whether ASDs fit into the most important recent trend of the pharmaceutical industry, continuous manufacturing.

In contrast with most fields of industry, batch technologies are prevailing in the pharmaceutical field due to the strict quality requirements and the Good Manufacturing Practice guidelines [11]. Lately, a number of experts and companies pay special attention to the continuous opportunities in the pharmaceutical manufacturing owing to the plenty of benefits such as robustness, environmental aspects, time and cost-effectiveness [12,13]. The FDA also sees great potential in the continuous processes because these methods can open new ways to the manufacturing of high-quality drug products [14]. Although only a few specific examples have appeared in the pharmaceutical market so far, continuous systems seem to be excellently applicable both in the manufacturing of active pharmaceutical ingredients and the preparation of final dosage form [15]. Among the marketed medicines manufactured with continuous technologies, Orkambi^TM^ and Symdeko^TM^ are especially interesting in the context of this review, since one of the APIs (Ivacaftor) in Vertex’s combination drug products possesses relatively weak dissolution properties and bioavailability in crystalline form; therefore, preparation of an ASD form by spray drying was applied to eliminate the above-mentioned disadvantages [4,16]. As a conclusion, advancements regarding ASDs are getting to play an important role in continuous processes as well. Nevertheless, there should be some cornerstones established if the ASD production technologies are attempted to fit into a continuous system. That is why an in-depth understanding of this topic may be of utmost importance.

The first part of the continuous manufacturing process of ASD-loaded formulations does not cause complications in general because the majority of the ASD preparation methods are continuous such as spray-drying or hot-melt extrusion (HME). However, challenges often appear after the production of ASD powders since the flow properties of these materials are quite poor in many cases. As a consequence, the feeding performance deteriorates, which could lead to drug content variation in the blends and also in the tablets or capsules [17]. These errors result in poor quality products in a batch process but even more in continuous technologies. Instead of accurate static weighing at batch production, precise determination of the mass flow needs to be achieved during continuous manufacturing. However, adjusting exact mass flows and controlling the mass flow ratio on the different feeders can mean huge challenges, especially in the case of powders with poor flowability. For this reason, improving the flowability of ASDs and enhancing the efficiency of the feeding is indispensable during continuous manufacturing to avoid the variation in the content uniformity.

A widely used method to increase the flow properties is granulation where it is important to bear in mind that ASDs are very sensitive to mechanical activation, high temperature and moisture, which can all cause the separation of the amorphous phases and possibly the crystallization of the API [18]. To prevent these, another solution could be the application of diverse excipients that ensure good flowability. Nonetheless, the selection of these materials is not so clear due to the possible incompatibility between APIs and excipients or bad flow properties of the blends. All of these have stimulated the technologists to be more engaged in the development of the feeders. Some recently published articles discuss the main issues and obstacles of continuous feeding, which draw special attention to the importance of the appropriate in-line monitoring and control systems as well [19]. During the feeding steps, the exploration of the relationship between material flow properties, feeder performance, and critical quality attributes could mean the basis for a suitable control system. Hence, investigating the effect of powder flow properties on the quality of the final product can be considered crucial.

Looking at the literature and the pharmaceutical market, both the development of ASD-based formulations and application of continuous technologies are emerging trends. Consequently, this review intends to collect the newest researches and the arising barriers connected with these two hot topics. Appraising the different ASD preparation methods, whilst taking into account the flow properties of the product, is an important part of this paper. Furthermore, feeder performances and the connected analytical and evaluation methods are also discussed.

## 2. Preparation of Polymer-Based Amorphous Solid Dispersions 

**Figure 1:**
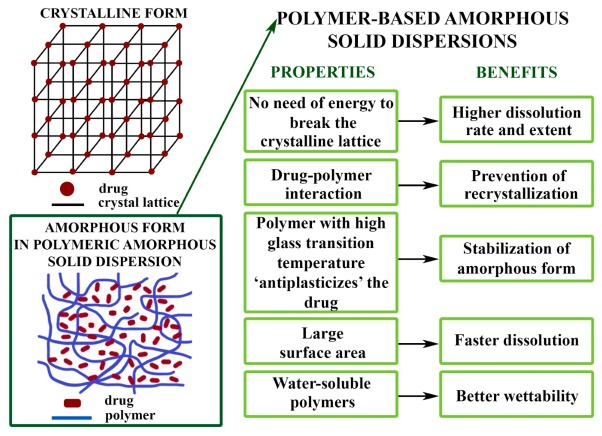
Advantages of drug-loaded polymer-based amorphous solid dispersions (ASDs).

Preparation and formulation of ASDs have evolved a lot since then not only concerning research and development but also in practical application. It can be stated that numerous pharmaceutical formulation publications are relating to drug-loaded, polymer-based amorphous systems due to their advantageous properties compared to the crystalline form (Figure 1). Although several impediments can be excluded by forming ASDs other, hitherto unknown pitfalls can emerge. One of the major troubles is that physical instability might turn up; therefore, recrystallization of the amorphous system befalls during storage [20,21]. This can be avoided for instance by choosing an appropriate polymer as a matrix that “antiplasticizes” the drug (raising its glass transition temperature) and/or forms intense interaction with the drug [9]. However, not only does the polymer influence the stability but the physicochemical characteristics of the drug, the ratio of the components and preparation methods also have an impact on the quality [22]. In conclusion, developers have to consider all of these factors during designing applicable ASDs.

ASD manufacturing techniques are divisible into two chief groups, namely solvent methods and melt or fusion methods. Versatile and applied-in-practice technologies can be listed in these two classes, which are compared and specified in many articles [4,23]. Spray drying and HME stand out all of the known ASD preparation methods because most of the commercially available ASD-based products are manufactured with these techniques. Their success can be explained with scale-up possibilities and by the fact that the process parameters are well-known and adjustable. Furthermore, spray drying and HME are both continuous technologies. Consequently, special attention is paid to these techniques in this review.

The most common solvent method is spray drying during which the solution is fed into a nozzle. After leaving the nozzle, the droplets are dried by counter- or co-flowing warm gas in the drying chamber. Then, the spray-dried form is collected through a cyclone into a collection bin [24]. In general, a powder of small particle size is obtained as the product at the end of this continuous technology [25]. One of the major advantages of this formulation is that faster dissolution can be achieved because of the enhanced surface area. In addition, there is no need for milling due to the fine powder products, which can also be considered beneficial. Although the poor flow properties of the spray-dried samples can be a significant drawback resulting in challenges in course of other continuous steps like feeding, blending, or tableting. At last, residual solvents could mean problems during scaling up thus their amount needs to be decreased as much as possible due to the strict quality requirements. In summary, many spray-dried products containing medicines can be found on the market and plenty of publications are referring to spray drying as a continuous process but a full continuous formulation of spray-dried products may have been examined only in case of the Orkambi^TM^ and the Symdeko^TM^ up to now.

Among the melt methods, the most prominent is HME adapted from the plastic industry [26]. During this process, the drug–polymer mixture is heated while the melt is transported through the extruder by one or two rotating screws (usually co-rotating screws). After leaving the equipment, the extrudates are arriving on a conveyor belt where the hot materials can solidify. Afterwards, the glassy product can be forwarded for cutting or grinding. This technique is also continuous and it has some benefits against spray drying such as solvent-free operation or better flow properties of the extruded powders [27]. However, only thermostable drugs can be formulated by HME, which is the greatest limitation of this method. Moreover, the poor compressibility of the products signifies a challenge in many instances; therefore, it also needs to apply fillers and other excipients if the tablet formulation is planned to be prepared by continuous manufacturing. Despite these detriments, many drug products consisting of extruded samples have been approved so far and continuous downstream processing systems, where HME is coupled with injection molding or 3D printing, have appeared in the literature recently [28,29].

During the years, some of the other ASD preparation methods were also investigated with regards to the generation of the final dosage form [18]. For instance, more examples have been published relating to tablets containing freeze-dried samples for improving the dissolution rate of poorly water-soluble APIs [30,31]. An alternative of conventional solvent evaporation methods can be the supercritical fluid processing where the quick evaporation can lead to residual solvent-free and free-flowing end product [32]. Based on the powder properties, tableting of the products can be fulfilled easily and this technique also can be implemented into a continuous line. Furthermore, electrospinning, a relatively novel technique for ASD preparation, also has to be mentioned as it seems to be a promising technology in the future based on the numerous publications in this field [33]. Related to the downstream processing of electrospun material, tableting was successfully performed from protein-type drug-loaded [34] and from acetaminophen-loaded electrospun fibers [35]. Since then, a minitablet formulation from electrospun nanofibers has been published in the literature [36], and tableting of itraconazole containing electrospun ASDs with other excipients also was scrutinized [37]. The latter one seems to be curiously promising in the industrial application because a scaled-up electrospinning method with high productivity and a rotary tablet machine were applied during the experiments. In addition, filling the fibrous materials into capsules is also a widely investigated area since it allows dissolution comparison between the novel formulation and marketed products [38,39]. However, most of the times the filling process or the tableting is accomplished manually due to the poor flow properties of electrospun materials. Consequently, the role of particle engineering and formulation steps is growing rapidly to achieve real industrial relevance of the electrospinning technology.

## 3. Continuous Pharmaceutical Manufacturing

### 3.1. The Current State of Continuous Production

From the beginning of the 2000s, some publications, crossing the batch-centered systems, started to deal with the possibilities of continuous technologies during pharmaceutical manufacturing [40,41]. After the first International Symposium on Continuous Manufacturing of Pharmaceuticals (ISCMP) [42], the researches and results related to this topic have become more and more popular, and the first FDA approved drug product (Orkambi^TM^) demonstrated the success of the new trend. The renovation of regulatory aspects became timely, which was one of the main topics on the second ISCMP in 2016 [43]. The white paper of the conference follows the previously accepted FDA and International Conference on Harmonization (ICH) guidelines [44,45,46,47] and on that basis, process monitoring and control have been emphasized peculiarly on the conference [43]. Summing up, batch technologies still dominate in the pharmaceutical industry but changing the mindset and opening towards continuous opportunities could denote significant advantages during the production of medicines [48].

As tablets are the most common final products, the majority of continuous downstream processing consists of feeding, blending, and tableting. Several companies came to the market with their continuous formulation line until today such as the GEA, Glatt, Lodige, LBBohle, Gericke/Gertise, and Bosch [49,50,51]. In the academic area, Simonaho et al., presented continuous manufacturing of tablets with the so-called PROMIS-line, which is a research and development continuous formulation approach established at the University of Eastern Finland [52]. This construction was tested in case of three different operations. The results demonstrated the applicability of all three configurations that can be implemented in a normal laboratory room. Nonetheless, the introduced system can be the basis of formulation studies, process parameter examinations and process analytical technology (PAT) measurements. Singh et al., studied continuous direct compaction of acetyl-para-aminophenol, silicified microcrystalline cellulose and magnesium stearate [53]. Since feeding has a high impact on the quality of tablets, the major influence factor, bulk density, was investigated. Near-infrared (NIR) spectroscopy was applied for real-time monitoring of powder bulk density by collecting spectra of the blends. For developing a NIR calibration model, powders with different bulk densities were measured. Afterwards, a control system was created using information from NIR spectra. This research proves that knowledge of critical quality attributes and those influencing factors are very important to obtain high-quality products. Experiments of Taipale-Kovalainen et al., also confirm the importance of crucial parameters [54]. Two different, long continuous manufacturing runs were tested during their research where the investigated technological set-up consisted of feeding, mixing, and tableting steps, these processes were connected by vacuum conveyors. The results present the intentional and unintentional deviations during long-term operation of continuous tableting.

Finally, it is worth mentioning in this section the end-to-end continuous manufacturing of pharmaceuticals. Its essence is to connect the synthesis of the APIs and formulation steps thereby a fully continuous system can be accomplished without human intervention. The first publication in this field, the work of the MIT’s researchers who achieved the synthesis and the purification of aliskiren-hemifumarate, then it was formulated to tablet form in a completely continuous manufacturing line in 2013 [15]. Later, four further APIs have been synthesized and formulated by using this technology to evidence its capabilities and success [55].

### 3.2. Continuous Formulation of ASDs

Numerous research papers exist in the literature with respect to continuous effectuation of the different formulation steps. Although the number of publications is limited relating to fully continuous preparation of ASD-based final drug products, it seems to be a quickly developing area.

Among the ASD formulation methods, HME is one of the most common techniques, which is basically a continuous technology. HME was used successfully several times to make amorphous drug-loaded pellets, granules or tablets [56,57]. Baronsky-Probst et al., studied twin-screw HME as an auspicious method for continuous production of pharmaceutical tamper-resistant tablets [58]. Potential critical process parameters of HME and their relationship with the critical quality attributes were investigated during their work. Fourier-transform NIR was applied as a PAT tool for detecting and controlling the influencing factors in real-time. Based on the results, the optimization of the HME technology could be facilitated by analyzing the process data and by using design of experiments (DoE). Interestingly, not only solid dosage forms can be prepared via HME but also gel formation, which prove the wide-range applicability of the technology. Pawar et al., investigated HME as a continuous opportunity for preparing topical diclofenac sodium gel [59]. In their research, the applied API was dissolved in water with other excipients and this solvent was injected into the extruder with a peristaltic pump. The other phase of the system was fed with a twin-screw volumetric feeder. These processes can be transformed into other continuous techniques as well. If the feeding of the pure, solid API seems to be difficult it might be worth feeding a “liquid masterbatch” while excipients with good flow properties can be fed with the commercially available feeders. In this way, the drug release was enhanced successfully and HME was used for continuous manufacturing of gel formulation at first.

Some publications in context to downstream processing present continuous systems where novel methods such as injection molding or 3D printing are connected to the HME for generating the final dosage form. Application of integrated HME-injection molding (IM) manufacturing platform was successfully developed for preparing sustained-release or immediate-release matrix tablets [28,60]. Although the dissolution properties of these drug-polymer systems seemed to be well controllable, the APIs were in the crystalline state in most cases where HME and IM were coupled. However, innovative 3D printing is another viable way to produce tablets after the HME process. Zhang et al., investigated the differences among 3D-printed tablets, directly compressed tablets from milled extrudates and tablets prepared from physical mixtures [29]. 3D printing is one of the state-of-the-art methods in formulation fields, which enables the design of complex dosage forms, thereby the production of personalized-dose medicines and immediate consumption products can be achieved. Linking HME and 3D printing resulted in extended acetaminophen release for every tested polymer. Furthermore, this article punctuates that combination of HME and 3D printing is considered advantageous for both bioavailability enhancement of the API and from the more effective production point of view. Similar conclusions were drawn in case of carvedilol-loaded 3D-printed floating tablets where four configurations were examined with different in vitro dissolution testing methods [61]. The major limitation of the 3D printing-based and integrated HME-IM formulation processes is that productivity is not comparable to the traditional tableting methods. HME coupled with 3D printing or IM seems to be an innovative and promising way of the personalized treatment but currently, it cannot be applied for mass production.

Formulation of amorphous drug delivery systems via spray drying is considered to be a continuous technology as well. The final dosage form of spray-dried samples can be inhalable dry powders [62] or oral solid dosage forms [63,64,65]. However, a totally continuous formulation line needs to start with the solution preparation and should take until the formulation of final dosage form, usually tablets. Consequently, technologists have to face similar challenges than in the case of HME, especially in the field of feeding. Cayli et al., prepared inhalation dry powders via spray drying where the API was fed in a suspension form while the mucolytic agent was added to it right before the ASD preparation process [66]. Continuous feeding of the API into the process seems straightforward if the API comes from flow synthesis and continuous purification (e.g., end-to-end manufacturing) [67,68]. However, continuous preparation of a feed solution from API powder using gravimetric feeding, separate solvent feeding and continuous mixing is much more challenging [69].

**Figure 2:**
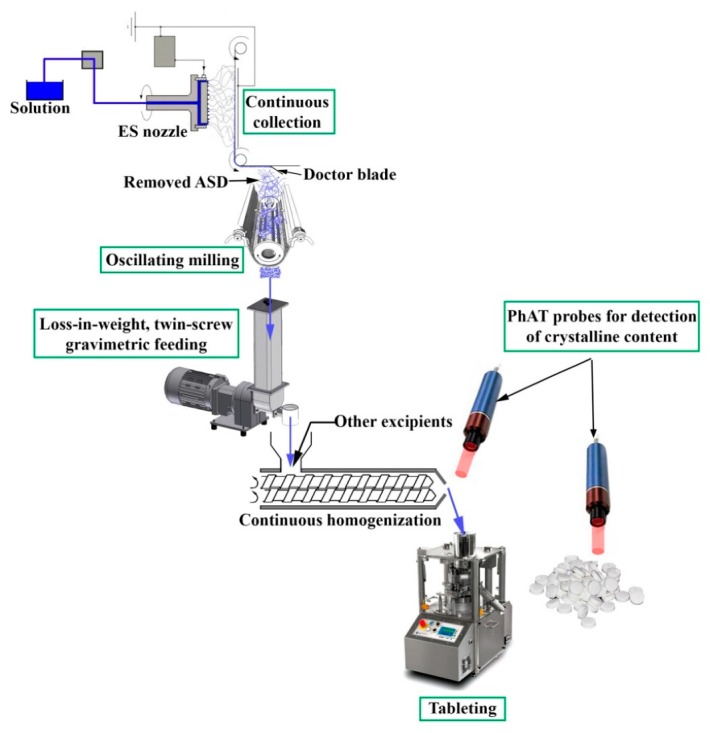
Schematic design of a possible electrospinning-based continuous formulation system [70].

A continuous formulation strategy was investigated in our earlier research article as well where ASD of spironolactone and polyvinylpyrrolidone-vinyl acetate copolymer was prepared with pilot-scale electrospinning [70]. Different part steps of a possible electrospinning-based continuous downstream processing seemed to be feasible such as the continuous collection of fibers, grinding, feeding, and tableting (Figure 2). Development of real-time analytical methods was also examined whereby detection of crystalline traces was efficiently implemented. This research highlights that both NIR and Raman spectroscopy can be applied for determining the absence of crystallinity. Nevertheless, the Raman-based models showed better predictability of crystalline traces, especially in case of powder blends. Therefore, it is worth examining the ‘amorphicity’ right after the electrospinning or after the homogenization.

Balogh et al., published a research paper in which continuous end-to-end production of an ASD-based solid form was accomplished [68]. During this work, electrospinning was introduced as an advanced linking technology between the synthesis of acetylsalicylic acid and its formulation into orally dissolving webs. Continuous preparation of the solution for electrospinning means the key step of connection flow reaction and electrospinning. If the polymer has no chemical interaction with the reactants, it can be added to the API solution during the synthesis. Otherwise, the polymer needs to be added right after the synthesis. Electrospinning allows the simple and quick removal of reaction solvents via fast evaporation. Therefore, crystallization, filtration, and drying can be excluded this way. In addition, continuous cutting and collection of the layered fibrous webs were successfully achieved by coupling Raman spectroscopy to the system.

Nevertheless, continuous preparation of tablets or capsules, the most popular solid dosage forms, could be important if the current batch systems are planned to be replaced. GlaxoSmithKline also developed their own continuous manufacturing method for preparing tablets where the process was approached from another side [71]. The speciality of the so-called liquid dispensing technology is that the tablets are pressed at first and then these placebo tablets are covered by the solution of the API and polymer or other excipients. This method is suitable to prepare low dose drug products in which the API is concentrated on the tablet surface. To confirm the exact amount of the dry dose, the surface of the tablets was examined by NIR imaging [72]. Although the research of Clarke and Doughty do not mention the “amorphicity” of the API on the surface of the carrier tablets the dosing solution contains a polymer and other excipients as well. The published method can consider being a platform technology for continuous film casting. Consequently, the technique can be applied for preparing low-dose ASD-loaded tablets in a continuous way if an appropriate drying process is chosen.

Designing the different continuous formulation steps is only one part of continuous manufacturing. Application of appropriate analytical methods is considered to be as important as the development of equipment with continuous mode. In the ASDs point of view, the quality control should focus on the crystalline traces of the API, which can be followed by in-line analytical methods as it was detailed earlier in case of electrospun samples. Furthermore, the above-mentioned NIR and Raman spectroscopy also was successfully applied for investigating the absence of crystallinity in spray-dried samples and extrudates [73,74,75,76,77]. However, there is another key factor besides the “amorphicity”, namely the amount of residual solvent, which needs to be determined during solvent-based ASD preparation methods. The earlier presented in-line spectroscopy methods can be used for this purpose as well. Tewari et al., successfully accomplished on-line monitoring of residual solvents during drying with a non-invasive infrared sensor [78]. Their results highlight that the conventional off-line analytical methods can be replaced with PAT tools to achieve continuous monitoring of residual solvents. A similar conclusion was deducted by Fonteyne et al., who investigated the moisture content during fluid bed drying [79]. The monitored drying system was part of a fully continuous from-powder-to-tablets production line where Raman and NIR spectroscopy is seemed to be effective to examine the fluid bed drying process in real-time. Consequently, the use of these methods can be satisfying for investigating ASDs as well, especially because fluid bed drying can be a continuous way for drying of ASDs [80,81]. Therefore, not only crystalline traces but residual solvents also can be examined with the same analytical technique with combining the evaluation methods.

## 4. Feeding as the Key Step During the Continuous Formulation of Solid Dosage Forms

Currently, solid dosage forms including capsules, tablets, and powders are the most frequent products on the pharmaceutical market [82]. Tablets and capsules are particularly popular products owing to their numerous advantageous properties such as accurate dosing, great patient compliance, easy mass production, and adequate storage possibilities. These are the main reasons why technologists strive to develop the mentioned formulations.

**Figure 3:**
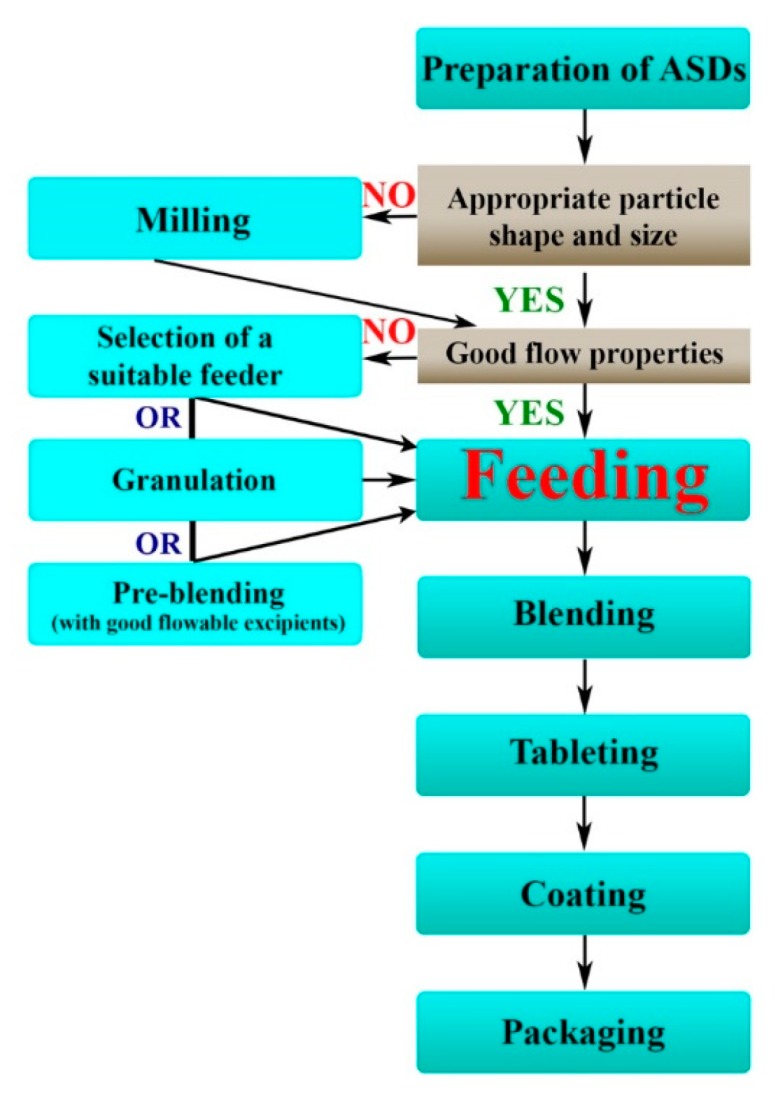
Flowchart of possible continuous pharmaceutical manufacturing routes for preparing ASD-loaded tablets.

The examples presented above give alternative ways for making ASD-loaded final drug products but the implementation of completely continuous formulation lines of ASDs presents many challenges in the case of the conventional way of tablet production. Figure 3 presents a flowchart of possible continuous pharmaceutical manufacturing routes for preparing ASD-loaded tablets.

Since flowability and feedability collectively affect the content uniformity during a continuous tablet formulation process, defining these two concepts is essential. While flowability has been described with different measurements for many years, feedability has a more complex character. Feedability was determined in the field of 3D printing as the mechanical suitability of the filaments for fused-deposit modeling [83]. The main principle is quite similar in the perspective of the pharmaceutical powders as well. As follows, the feedability can be defined as the mechanical aptitude of solid raw materials for reliable and precise dosing. It means, if the powder is characterized with good feedability, it can be dosed easily, uniformly and accurately. Although flowability greatly influences the feeding, feedability also depends on the properties of the applied feeder and the adjusted parameters. Consequently, feedability has to be described not only with the powder properties but with other factors as well. For this purpose, data from a catch scale are collected in general to calculate the feed rate, the feed factor, different moving averages and relative standard deviations, which express the feedability very well [84]. The so-called catch scale displays the pure gravimetric signal without any pre-treatment thus it enables the comparison of the feeder performances. This way it can occur that the flowability of the investigated powder is poor but using a suitable feeder, the feedability will be appropriate according to the calculated features.

On the other hand, handling of the poorly flowable powders can be facilitated via granulation or by using excipients with good flow properties. In the context of ASDs, HME can be the most obvious method to simultaneously increase the dissolution properties and the flowability of APIs [57]. A good opportunity can be the application of other commonly used wet or dry granulation methods. However, there are some extra challenges during the granulation of ASDs with these widely used techniques. For instance, mechanical strength during roller compaction or moisture content during wet granulation has a huge impact on the stability and on the release of the amorphous API; therefore investigating the trace crystallinity is very important in these cases. Successful roller compaction of evacetrapib-loaded ASDs and wet granulation of indomethacine-loaded ASDs proves the feasibility of the granulation processes in this field [85,86]. However, it is important to note that a feeding step is also needed before continuous granulation thus this kind of flowability enhancement is usually used before the tableting and not before the blending [87]. The third option can be the pre-blending of the poor flowable materials with excipients, which usually means a semi-continuous processing step during tablet manufacturing. For the above-mentioned reasons, granulation and pre-blending are worth using only in case of powders with extremely critical flow properties. If it is possible, selection of an appropriate feeder can be the most convenient way.

### 4.1. Powder Characterization

Powders can be typified with a myriad of descriptors, which are greatly related to their behavior and these values can provide useful information for designing the formulation steps [88,89]. Hlinak et al., made a really clear overview of the critical material properties and their effect on the product attributes and the processing behavior [90]. Based on their overview, Table 1 highlights the possible critical attributes connected to the flow. Moreover, it includes the effects of the listed parameters on the powder flow and it reveals the possible measuring methods as well. If characteristics of the materials get clear by using the appropriate analysis techniques, the next outstanding point is to find the correlation between the measured properties and feedability for building up a continuous system of good quality. Since more companies deal with the fabrication of different feeders [91,92], knowledge of material flow properties could be especially handy when choosing the appropriate feeder peculiarities. This is supported by the research of Wang and co-workers who predicted feeder performance based on material flow properties [93]. Ultimately, it can be claimed that investigating the impact of all the above-noted variables on the diverse processing steps needs a high amount of the given powder, which is not preferable during the early drug development or in case of costly samples. Recently, a research article was published about a multivariate raw material property database that can provide a solution for handling the problem of high powder demand [94]. Fifty five different raw materials inclusive of APIs and numerous distinct excipients were characterized. As a result, this dataset can facilitate the development of formulations and pharmaceutical dry powder processes became designable by building predictive models based on the descriptors. Bostijn et al., gave an excellent example to the applicability of this database via investigating the feeding of 15 different powders (including APIs) by low feeding rate [95]. The main conclusion of the article was that less feeding experiments would be necessary by following the presented approach and thus, the raw material consumption would also decrease during new drug development. Furthermore, these datasets can be easily transported to the continuous formulation of ASDs if their powder characteristics are similar to one of the included APIs or excipients in the database. Another attractive solution is the simulation of the different processing steps in order to avoid the wasting of expensive raw materials during the early development phase. The research of Boukouvala and Ierapetritou demonstrated that the costly flowsheet models can be simplified via surrogate-based optimization (application of another powder with similar peculiarities instead of the expensive API) [96].

### 4.2. Flowability of ASDs

**Figure 4:**
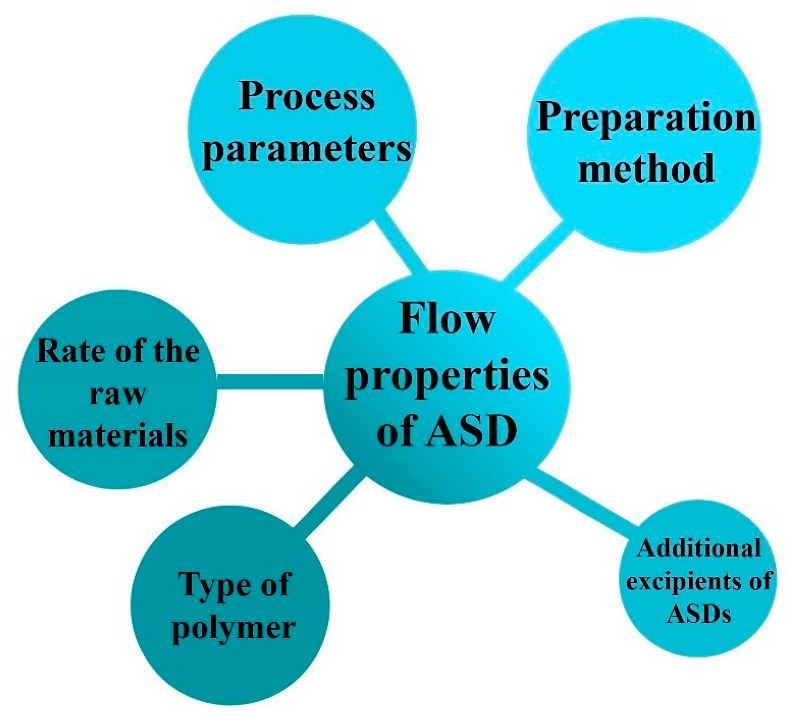
Main influencing factors of polymer-based ASDs’ flowability.

Powder flow properties have an impact on downstream processing of polymer-based ASDs as well. Inadequate flowability can cause bridging, arching, or rat-holing during tableting, which deteriorate the processing time and the efficiency of die filling [116]. Furthermore, powder behavior also influences the feeding in case of continuous manufacturing and thus, successful implementation of this step strongly depends on the physical properties of the ASDs and on the external variables such as consolidation, aeration or the applied equipment [89]. ASDs can be characterized with bad powder flow properties in many instances; therefore, automatic and continuous mass production of ASD-loaded tablets may encounter obstacles [18]. The major influencing factors of polymer-based ASDs’ flowability are summarized in Figure 4. As the different preparation methods and process parameters result in various kinds of products, the flow properties differ accordingly. In addition, to a lesser extent but the type and amount of the applied raw materials also determine the flow characteristic of the produced ASD. Finally, flowability can be influenced by adding extra excipients to the composition before the ASD preparation.

Davis et al., studied the impacts of spray drying and HME on powder flow, compression and dissolution in case of itraconazole-containing ASDs [117]. A ternary ratio of API-Soluplus^®^-HPMCP 30-40-30 (*w*/*w*%) was selected for preparation of ASDs. The different samples were analyzed by scanning electron microscopy and Freeman FT4 powder rheometer to receive information about the particle size, morphology, and the powder flow, respectively. In terms of powder flow, spray-dried materials had poorer characteristics than milled extrudates due to the smaller particles sizes, more complex morphology, and cohesiveness. Decreasing the particle size of extrudates is possible too, by different intensive milling processes [118]. Although the dissolution properties of the extruded materials can be improved with reducing the size ranges then the same flowability problems can occur as by the spray-dried samples in general [119]. Poor flowability of spray-dried samples has industrial relevance as well. The flow properties of etravirine-loaded spray-dried ASD needed to be improved by continuous roller compaction together with microcrystalline cellulose during the downstream process [120].

In the flowability point of view, fibrous ASDs can be especially disadvantageous since the longitudinally macroscopic but microscopic width structure results in fluffy, low bulk density (usually between 0.01 and 0.1 g/mL) powders. For this reason, the grinding of the fibers might be needed to improve the flow properties of the samples [121]. Furthermore, there are several milling methods, which are considered to be continuous such as conical milling or oscillating milling. Therefore, the electrospinning-based formulation steps can be transformed easily into continuous processes if the electrospun material is grindable. Different sugars turned out to possibly have an impact on the friability of polyvinyl alcoholnanofibers thus the application of extra excipients can help in case of electrospun materials as well [122].

### 4.3. Improvement of the Flowability of ASDs for Continuous Feeding

**Figure 5:**
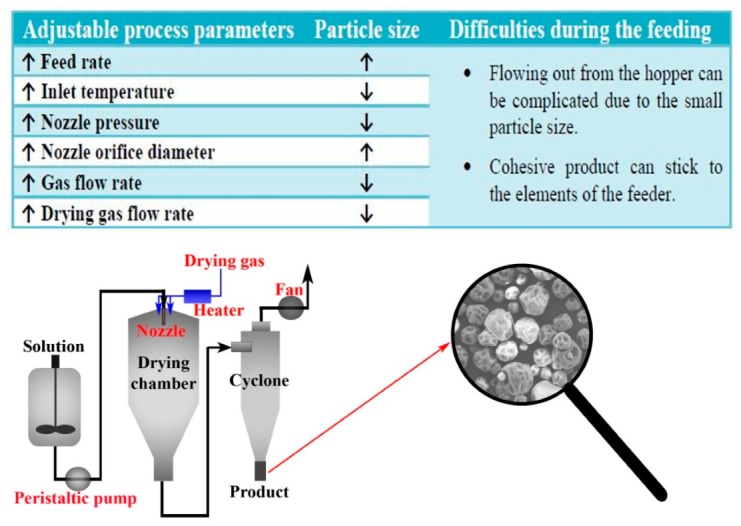
Effects of process parameters on the particle size of the product (red bold labels indicate the influencing factors.).

The morphology of the spray-dried materials and thus the flow properties can vary according to the adjusted values on the spray dryer (Figure 5) [123]. Regarding this, Vehring et al., highlight in their experiments that particle formulation during spray drying is strongly influenced by the relationship between surface recession and diffusion of the solutes [124]. Consequently, changing the components and therefore the viscosity of the starting solution will have an effect on the morphology of the products at the same conditions. This observation is also supported by Goddeeris and Van den Mooter who prepared free-flowing spray-dried samples [125]. The aim of their research was to maximize the dissolution extent of a model API, which was successfully performed by adding a surfactant (d-α-tocopheryl polyethylene glycol succinate 1000) to the polymer–drug composition. However, Carr’s index and the Hausner ratio decreased with an increasing amount of the surfactant, which indicates better flowability. This can facilitate the downstream processing later on. Ramesh et al., also added an extra excipient, microcrystalline cellulose, to the solution before spray drying, which was suitable to increase the flowability of the spray-dried samples [126]. Another widely-used method for improving the flow properties of the spray-dried particles is the roller compaction [127]. For instance, Angi et al., were applied dry granulation to reach better flowability and reduce the electrostatic charging of the small particle-size samples prepared by spray drying [128]. The spherical shape of the samples did not change after the granulation process but large agglomerates were visible in the scanning electron microscopy images. These agglomerates seemed to be favorable in the flowability point of view since the granules were characterized with better flow properties according to the Hausner ratios and Carr’s indices.

A conclusion similar to the previous examples can be stated in the case of materials prepared by the electrospraying method [129]. During this research, bulk and tapped densities increased with higher poly(lactic-*co*-glycolic acid) concentration. The authors also reveal the importance of process parameters such as voltage, feeding rate, and the polymer concentration, which are key factors in the formulation of particles.

In the case of fibers prepared with electrospinning, grinding is indispensable to handle the electrospun material in the further formulation steps. Development of grindable fibers improved the flowability of protein-type drug-loaded electrospun samples and thus it facilitates the tableting process [121]. However, low bulk density characterizes the electrospun fibers after grinding in the most cases thus pre-blending with a good flowing excipient, such as large particle size microcrystalline cellulose, seemed to be necessary to improve the efficiency of continuous feeding of spironolactone containing fibers [70].

The presented examples point out that designing of reliable feeding with ASDs to a continuous formulation line could be extremely challenging due to the poor flowability and low bulk density of the powders (especially in case of solvent techniques). For this reason, it is worth to take an outlook for ASDs in context with feeding and surface modification as well. It is important to note that ASD samples with smaller particle size (usually the ASDs prepared by solvent techniques) have quite similar flow properties to the micronized APIs in general thus the handling and the downstream processing need the same considerations as by APIs with poor flowability [130]. Consequently, continuous dry coating, such as fluid energy milling (FEM) or the use of Comil^®^ for in situ coatings with glidant (for example, Aerosil^®^), can be promising surface modification methods to improve the flow properties of ASDs, too [131,132]. In the literature, only a few examples can be found about surface-modified ASDs among which in situ surface modification of spray-dried samples seems to be a potential solution [133]. The research of Elversson and Millqvist-Fureby pointed out that adding suitable excipients in an appropriate amount to the solution results in products with good flowability. If the in situ way is not feasible due to solubility differences, the previously mentioned dry-coating methods can be used (Comil^®^ or FEM) because these techniques are easily insertable into continuous manufacturing processes. In addition, these systems are suitable not only by spray-dried products but in the case of other solvent-method prepared ASDs. For instance, electrospinning could be an alternative to freeze-drying while the continuous collection of fibers also seems to be feasible using a cyclone [134]. However, powder properties of the electrospun samples are fairly weak, which can make the formulation process difficult. Therefore, the milling of these products might be needed if tablet forms are planned to be achieved [37]. On the other hand, other formulation strategies such as dry coating or granulation also may be appropriate to increase the flow properties of the fiber-based powders.

In addition, surface modification is also applicable for decreasing the electrostatic charging of the powders to avoid the charge generation during the formulation processes. Jallo et al., investigated magnetically assisted impaction coating as a potential method to improve the flow and decrease the electrostatic charging of micronized acetaminophen [135]. During their research, a clear correlation was observed between the flow and the electrostatic charging of the uncoated and dry-coated micronized API. Electrostatic charging can also occur with ASDs due to the micro- or nano-size particles or if the applied polymer easily charges electrostatically. The powders can stick to the wall or to the screw of the feeder in case of these kinds of ASDs, which cause high relative standard deviation in the feeding rate.

Taking together, these methods would seem to suggest that increasing the flowability of bulk powders could be accomplished without using the well-known granulation, too. Nevertheless, a feeding step should always be inserted before the granulation during continuous manufacturing lines and thus, particle engineering and surface modification are especially significant by designing continuous formulation lines of ASDs.

### 4.4. Considerations Related to the Feeders

#### 4.4.1. Feeder Peculiarities

Another side of the feeding efficiency is feeder characteristics that can be modified to enable faster feed rates and more reliable weight variation. Different evolving, shape, and size of the feeders offer a variety of applicability to weigh continuously the diverse pharmaceutical blends. For instance, Coperion K-Tron, Brabender Technologie, or Técnicas de Alimentación Dinámica (TAD) fabricate manifold pharmaceutical and other feeders for handling most bulk materials in a wide range of applications [136,137,138]. To overview the opportunities, Table 2 presents different Coperion K-Tron feeders and suggestions in connection with the selection of the feeders for various powder types. It can be stated that many feeding considerations apply to the designing of the tableting process, application of powders with various bulk densities, or feeding between the downstream processing steps. In light of this, a large number of variables can arise that is why understanding the material behavior and the capability of the feeders are very important.

**Figure 6:**
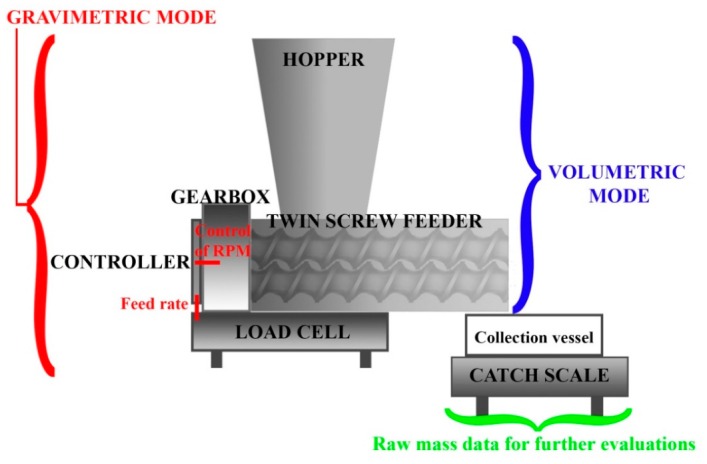
Schematic design of a possible loss-in-weight feeder setup with different operation modes.

Most commonly used pharmaceutical feeders operate on the principle of loss-in-weight (LIW) and mostly consist of one or two screws (Figure 6). Weighing platform (load cell) is the inherent part of this type of feeders because it ensures the ability to control the feed rate. Basically, LIW feeders can be used in two different modes. The simpler operation is the volumetric mode whereby the moving elements have constant rotation speed. In contrast, the gravimetric method applies the signal of the load cell to control the rotation of the screws based on the mass flow. This unit operation can handle flow variability caused by bulk density changes relating to the emptying of the feeding hopper [139,140]. As a consequence, gravimetric feeding is a promising method in case of ASDs as well since the bulk densities can vary in a wide range, especially if different size agglomerates are prepared during granulation processes. In addition, a catch scale is usually utilized to gain data from the outlet of the feeder, which give useful information about the feedability and it could also have a significant contribution to the feedback control [84].

Variations of the LIW systems differ in the type of the moving element, which can be a screw, rotating cell, belt, or vibratory channel. The latter three were not too widespread in the pharmaceutical field so far for dosing APIs, excipients or blends. For instance, a vibratory feeder was rather applied for measuring powder flow by using a small amount of the materials [141]. However, vibration systems are more common in the food and agro-industry for feeding bulk solids accurately. Ola and Popescu published a quite handy article relating to the adjustable parameters during vibratory feeding [142]. According to their results, the vibrations’ amplitude, the amount of the dosed powder from the hopper and the material thickness on the vibrating tray has a great impact on the precision of the feeding. Furthermore, vibratory feeding was found to be describable with virtual models as well, which may be very helpful in the design of new equipment with high accuracy for continuous processes [143]. The presented considerations can be translated into pharmaceutical powders for dosing reliably both at high and low performance even for problematic materials. The most common way in the pharmaceutical industry is to use twin-screw feeders where it is worth changing the type of the screw according to the applied material [92]. Since a lot of polymer-based ASD prepared by solvents method can be described as a sticky powder, a fine concave screw would be the best choice for precise feeding. Based on Table 2, vibratory feeders can be applied in case of most types of powders thus these devices seem to be promising in case of ASDs as well.

#### 4.4.2. Importance of Feeder Selection

Polymer-based ASDs can be quite sticky or fluffy materials, which is why the selection of a good feeder may mean a solution to handle the bad powder characteristics of these samples. Engisch and Muzzio published several papers in connection with loss-in-weight feeders. One of their work introduces the characterization of a K-Tron KT35 loss-in-weight feeder with different screws by using three distinct powders [84]. Both volumetric and gravimetric modes of the feeders were examined while the feed rate was monitored. Feeder tooling, powder, and speed had a significant impact on feeder performance according to the analysis of variance of the feeder characterization. Eventually, the authors suggested that predictive models could be developed if a database of feeder performance and powder behavior was prepared. Similarly, the LIW feeding of a poorly flowing API from the aspect of twin-screw granulation process was studied [91]. Different LIW gravimetric feeders were tested during these experiments to achieve 75% API content in the granules. The results evidenced that the rational selection of the feeder determines the success of the continuous twin-screw granulation. In another study of Engisch and Muzzio, various powders including an API, excipients, and a lubricant were fed by suitable types of feeders for the given materials. Different screws and screens were tested in case of each feeder and feeding performance was typified by the feed rate. This research presents a possible way for the proper feeder selection in case of different powders [92]. Although continuous feeding of poorly flowable, usually micronized APIs means a challenge in itself but the feeding of low-dose APIs is even more difficult. A vibratory feeder proved to be suitable for micro-dosing of two powders while the periodicity of the fluctuation was changed [144]. Besenhard et al., not only investigated the micro-feeding but also studied the micro-mixing via a computational simulation method.

Besides the material flow properties, the feed rate deviations caused by hopper refill are also fairly important in a practical application point of view [145]. During a continuous process, hopper refill has to be solved while the feeder is operating. Type of refill method or its frequency can strongly influence the feed rate deviation. According to the scheduling of the refill, the lowest standard deviation was observed when the hopper was refilled from 60% to 80% level. Furthermore, the applied material also has an effect on the feed rate deviation, which was investigated by comparison of acetaminophen and zinc oxide.

The next step in this area is the control of the feeder, which was successfully accomplished by Joshua Hanson [146]. During this research, three feeding failures (excessive mass flow variability at steady operation, inaccurate feeding relative to set point and special cause transient disturbances) were investigated in loss-in-weight feeding. Based on the results, the use of ratio control was found to be an effective approach to improve feeder performances. In this way, there is no need to precisely adjust the mass flow of the poorly flowable APIs or other powders. Monitoring the ratio of each mass flow allows the feedback-control of the feeder of the well flowable excipients. Since the feedability of powders with good flowability is easier, the controlling could be simpler and faster. In the case of ASDs, the above-detailed methods would be especially useful because the blending process could be predictable and controllable based on a well-monitored feeding step. In addition, feeding failures also have a higher chance due to the poor flowability and feedability of ASDs.

## 5. Conclusions

Increased interest in continuous technologies and a growing number of ASD-loaded drug products raise the issue of the joint examination of the two areas. ASDs enable the development of poorly water-soluble drug candidates into final dosage forms while the dissolution of the API improves and thus, greater bioavailability could be achieved in many cases. Downstream processing of these materials is getting more attention not only in the academic area but also in the industry. However, published articles relating to the continuous formulation of ASDs confirm that it is quite a new aspect in the pharmaceutical field. The majority of the research groups attempt to find alternative ways instead of the conventional formulation process of tablets such as combination HME with IM or 3D printing. Although these solutions avoid the barriers caused by the poor flowability of ASDs, the scalability is limited in most cases. In contrast, the traditional formulation line is more suitable for mass production thus improving the flow properties is a hot topic at present. As it was presented in this review, flowability can be enhanced by various methods and feeder marketed companies offer more and more expedient equipment as well. Considering all this, continuous downstream processing of ASD is worth investigating closer in the future to get more reliable processes and products.

## Figures and Tables

**Table 1:** The potential impact of powder properties on flow.

Property	Impact	Measuring	Ref.
Particle size and shape distribution	Flowability increases with increase in particle size; Spherical shape results in favorable flow properties	Sieve tower; Microscopy and image analysis; Scanning electron microscopy; Laser diffraction	[97,98,99]
Bulk density	Hausner ratio and Carr’s index can be determined based on bulk and tapped densities; With increasing Hausner ratio, the flowability decreases; Decreasing Carr’s index means an increase in the flow	Tapping machine; Dynamical tap density tester; Powder rheometer	[100,101]
Surface area	With increasing specific surface area, the flowability decreases in general	Pycnometer;	[102,103]
Surface energy	Increased surface energy leads to poor flowability	Inverse gas chromatography for separation and mass spectrometry for detection	[104,105]
Flow	Higher flow rate indicates higher flowability	Flow through an orifice	[106]
Cohesiveness	High cohesiveness allows the powder bed to be compressed easily and the flowability is poor; The cohesive index quantifies the extent of deviation from an ideal conically shaped heap	Powder rheometer; Granular material heap analyzer	[107,108]
Internal and wall friction	Effective angle of internal friction influences many aspects related to flow behavior, e.g., risk of arching and risk of segregation due to unwanted flow patterns; The higher the wall friction angle the more difficult it is to move the powder along the wall surface (the worse the flowability)	Shear cells	[109,110]
Static charge	Static charge compromises the free-flowing of the powders	Charging device; Faraday cup; Granular material electric charge analyzer	[111,112]
Hygroscopicity	Lower hygroscopicity results in better flowability	Dynamic vapor sorption; Loss on drying	[113,114,115]

**Table 2:** Application opportunities of Coperion K-Tron feeders [106].

Feeder Type	Powders
Loss-in-Weight Belt Feeders	Fragile products; Powders with special characteristics
Vibratory Feeders	Fragile ingredients; Fibers; Glass fibers; Rough-grained powders; Granules
Bulk Solids Pump Feeders	Free-flowing pellets; Granules; Flakes; Friable products
Twin Screw Feeders	Sticky, bridging or flooding powders; Fibers; Glass fibers
Single Screw Feeders	Pellets; Other free-flowing bulk materials
Smart Weight Belt Feeders	Large volume of powders with different flow characteristics
